# Monitoring of Pathogen-Specific T-Cell Immune Reconstitution after Allogeneic Hematopoietic Stem Cell Transplantation

**DOI:** 10.3389/fimmu.2013.00276

**Published:** 2013-09-17

**Authors:** Shigeo Fuji, Markus Kapp, Hermann Einsele

**Affiliations:** ^1^Department of Internal Medicine II, Division of Hematology, University Hospital of Würzburg, Würzburg, Germany; ^2^Division of Hematopoietic Stem Cell Transplantation, National Cancer Center Hospital, Tokyo, Japan

**Keywords:** virus, fungi, T cell, immune reconstitution, allogeneic stem cell transplantation

## Abstract

The clinical outcome after allogeneic hematopoietic stem cell transplantation (HSCT) has been significantly improved during the last decades with regard to the reduction in organ failure, infection, and severe acute graft-versus-host disease. However, severe complications due to infectious diseases are still one of the major causes of morbidity and mortality after allogeneic HSCT, in particular in patients receiving haploidentical HSCT or cord blood transplant due to a slow and often incomplete immune reconstitution. In order to improve the immune control of pathogens without an increased risk of alloreactivity, adoptive immunotherapy using highly enriched pathogen-specific T cells offers a promising approach. In order to identify patients who are at high risk for infectious diseases, several monitoring assays have been developed with potential for the guidance of immunosuppressive drugs and adoptive immunotherapy in clinical practice. In this article, we aim to give a comprehensive overview regarding current developments of T-cell monitoring techniques focusing on T cells against viruses and fungi. In particular, we will focus on rather simple, fast, non-labor-intensive, cellular assays which could be integrated in routine clinical screening approaches.

## Introduction

The clinical outcome after allogeneic hematopoietic stem cell transplantation (HSCT) has been significantly improved during the last decades with regard to the reduction in organ failure, infection, and severe acute graft-versus-host disease (GVHD). These advances have rendered allogeneic HSCT to an integral part of treatment for hematological malignancies (1, 2). However, severe complications due to infectious diseases are still one of the major causes of morbidity and mortality after allogeneic HSCT, in particular in patients receiving haploidentical HSCT or cord blood transplant due to a slow and often incomplete immune reconstitution. The reduction of immunosuppressive drugs could pave the way to strengthen T-cell responses against pathogens after allogeneic HSCT. However, blind rapid tapering or cessation of immunosuppressive drugs is associated with an increased risk of alloreaction with subsequent clinical consequences such as increase of severe acute or chronic GVHD as demonstrated previously (3, 4). In order to improve the immune control of pathogens without an increased risk of alloreactivity, adoptive immunotherapy using highly enriched pathogen-specific T cells offers a promising approach. Adoptive immunotherapy against several pathogens has been already evaluated within clinical trials as reviewed previously (5).

In order to identify patients who are at high risk for infectious diseases, several monitoring assays have been developed with potential for the guidance of immunosuppressive drugs and adoptive immunotherapy in clinical practice. In this article, we aim to give a comprehensive overview regarding current developments of T-cell monitoring techniques focusing on T cells against viruses and fungi. In particular, we will focus on rather simple, fast, non-labor-intensive, cellular assays which could be integrated in routine clinical screening approaches.

## The Role of Pathogen-Specific Immunity in Prevention and Control of Infectious Diseases

### Virus-specific T-cell immunity

It is well-known that virus-specific T cells are important to prevent and/or control viral infection after allogeneic HSCT. Cytomegalovirus (CMV) is one of the most intensively investigated targets of immunotherapy after allogeneic HSCT (6). After allogeneic HSCT, the first emergence of CMV reactive antigenemia triggers the expansion of donor-derived CMV-specific T cells. These expanded cells usually have a phenotype of effector- or effector-memory type. The presence of CMV-specific T cells in patients after allogeneic HSCT was reported to be protective against the recurrence of CMV antigenemia (7–12). Especially, CMV seropositive patients with profound immunosuppression or CMV seropositive patients who received stem cells from a CMV-seronegative donor are at high risk for a significant delay in reconstitution of functional CMV-specific T cell which is associated with persistent CMV viremia and a higher risk of CMV disease (8–12). Furthermore, adoptive T-cell therapy of CMV-specific T cells was demonstrated to be effective for the prophylaxis and treatment of CMV disease after allogeneic HSCT (13, 14).

The importance of virus-specific T cells has been also demonstrated with regard to other viruses such as adenovirus (15–18), EB virus (19–21), BK virus (22–25), and JC virus (26, 27). Recently, banked third party virus-specific T cells were reported to be safe and effective for the treatment of viral disease after allogeneic HSCT, which circumvents a major obstacle to the wider use of virus-specific T cells, in particular in patients after cord blood transplant (28).

The monitoring of T-cell immunity against these viruses can be useful to assess the risk of viral infections. The benefit of adoptive T-cell therapy as prophylaxis or as treatment should be ideally assessed in prospective clinical trials.

### Fungus-specific T-cell immunity

For a long time fungus-specific T cells have not been regarded as important to control fungal diseases. However, there is growing evidence that CD4^+^ T cells provide defense mechanisms against fungal infection (29–32). The majority of patients diagnosed with invasive aspergillosis after allogeneic HSCT are not neutropenic which for a long time was considered the only or at least the most important immune mechanism to prevent fungal disease (33, 34). Recent studies have shown that fungus-specific T cells are detectable in healthy individuals and patients with hematological malignancies (29, 32, 35). Due to the paucity of clinical studies which assessed the impact of presence of fungus-specific T cells compared to virus-specific T cells, further prospective studies which assess the importance of fungus-specific T cells on preventing/controlling fungal infection are urgently needed.

In addition, the improved outcome of invasive *Aspergillus* following adoptive T-cell therapy for invasive Aspergillosis demonstrates the clinical value of transfer of fungus-specific T cells from the stem cell donor (36). Furthermore, recent reports showed that the GMP-grade-Aspergillus-specific T cells could be produced for clinical trials using commercially available enrichment protocols (37–40).

## How Can Pathogen-Specific Immunity be Monitored?

Up to date, various methods are available to assess T-cell immunity against specific antigens. However, some methods like limiting-dilution assays are not feasible due to the labor-intensive works which cannot be a part of routine clinical practice. Here we summarize three simple broadly available methods which can be performed using peripheral blood mononuclear cells (PBMC) or whole blood without long-term *ex vivo* culture, and using commercially available reagents. In addition, PBMC can be frozen without the loss of the function when tested in intracellular cytokine staining (ICS) or Enzyme-linked immunosorbent spot (ELISPOT), which is practically very important with regard to reproducibility and standardization with strict quality control (41). Combinations of these assays are needed for the confirmation of results and comprehensive measurement of different T-cell functions. The advantages and disadvantages of each method are summarized in Table 1.

**Table 1:** **Comparison of three T-cell assays**.

Assay	Advantage	Disadvantage
ELISPOT	No cell fixation	Cell of origin of cytokine production unclear
	The same cells can be retested	No sorting of cytokine-secreting cells possible
	Suitable to test many samples simultaneously	
	Cytotoxicity assay can be induced	
	A lower number of cells required for analysis	
Intracellular cytokine staining	Assessment of multiple cytokines at single cell level	Cells have to be fixated and permeabilized
	Combination with phenotyping and cytotoxicity assay	No sorting of vital cell populations possible
MHC-multimer staining	Combination with phenotyping Sorting of antigen-specific T cells, which can be used for adoptive T-cell therapy Detection of dysfunction/non-functional antigen-specific T cells, e.g., naïve T cells	Each tetramer has to be produced for respective HLA typing and peptide
		Not suitable for the assessment of cytokine secretion (functionality)
		

### Enzyme-linked immunosorbent spot

**Figure 1:**
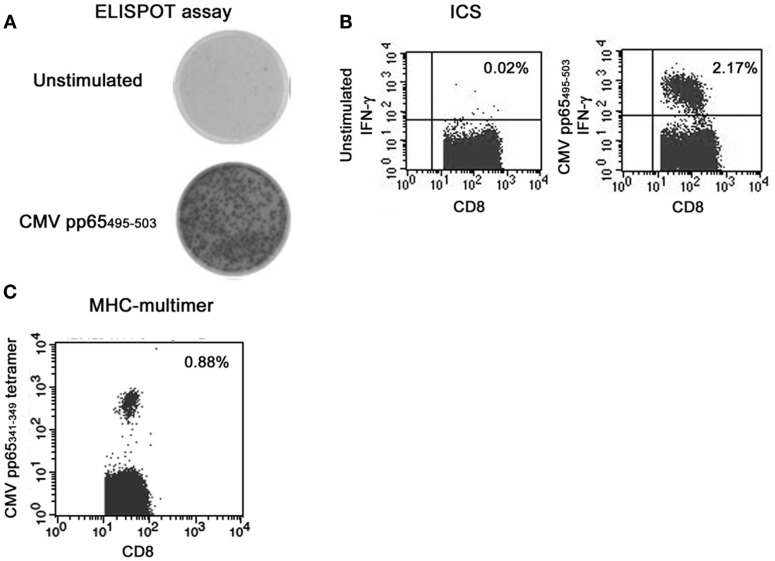
**Representative results of immune monitoring of CMV-specific T cells after allogeneic hematopoietic stem cell transplantation (A) ELISPOT assay, (B) intracellular cytokine staining, (C) tetramer**.

Enzyme-linked immunosorbent spot is one of the most established methods to detect functional immunity (42–44). In brief, PBMC are cultured for 18–24 h on an anticytokine capture antibody-coated membrane in the presence of an antigen. Following culture, each antigen-specific T cells will release cytokines that will bind to the capture antibody on the membrane. The cells are then washed and the secreted cytokines can be detected on the membrane by use of an enzymatically labeled antibody and insoluble chromogenic substrate. In this assay, frequencies of cytokine-secreting T cells can be counted after *in vitro* stimulation of PBMC by defined antigens/peptides without previous *ex vivo* expansion. In addition, ELISPOT assays allow the size and intensity of the spots to be calculated, which correlated with the amount of cytokines secreted by each cell. As shown in Figure 1A, we are able to detect the induction of IFN-γ after the stimulation with CMV pp65 IE-derived peptides in patients after allogeneic HSCT.

Enzyme-linked immunosorbent spot offers several advantages: (1) many samples can be tested simultaneously using one plate; (2) the secretion of cytokines can be assessed in contrast to the artificially retained cytokines in ICS; (3) the cell numbers can be downscaled per well in comparison to flow cytometry-based methods.

Cytotoxic activity can be assessed using granzyme B ELISPOT. Granzyme B ELISPOT has been reported to have excellent correlation with the ^51^Cr-release assay for measuring cytotoxic activity of T cells (45, 46). Furthermore, multiple-color fluorospot assays make the analysis of single cells secreting several cytokines possible (47, 48). Detecting each cytokine with a different fluorophore, polyfunctionality of T cells can be analyzed, suggested to be important to protect against various infectious diseases.

The disadvantages of ELISPOT are: (1) it is difficult to determine which immune cells secrete IFN-γ. This is critical to assess the immune status after allogeneic HSCT. As Wang and Colleagues reported, the response to 9-mer peptide, which is expected to induce cytokines in an HLA class I-restricted, can also be HLA class II-restricted (49, 50). Therefore, when IFN-γ induction in ELISPOT assay is detected using stimulation with lengths of peptides including peptide-pool, cell of origin of IFN-γ secretion has to be determined using CD4/CD8 depletion or HLA blocking assays; (2) sorting of cytokine-secreting cells is impossible.

### Intracellular cytokine staining

Intracellular cytokine staining is also one of the most established methods to detect functional immunity (51, 52). In brief, PBMC are cultured for 6–18 h in the presence of an antigen. To preserve the generated cytokines within the cytoplasm, a Golgi-blocking agent (e.g., Brefeldin A or Momensin) is added during the stimulation. After the stimulation, samples are collected, fixated and permeabilized. Consecutively, antibodies against intracellular cytokines are added. When surface markers whose binding is sensitive to fixation and permeabilization are stained in combination with ICS, they should be stained before fixation and permeabilization. Stained cells were analyzed using a flow cytometer. A representative result is shown in Figure 1B.

The advantages of ICS are as follows: (1) the phenotype of each cell which secretes the cytokine can be determined (53); (2) the cytolytic potential of the target cells can be assessed using CD107a degranulation assay in combination with the assessment of multiple cytokine induction.

The disadvantages of ICS are: (1) reagents such as Brefeldin A are required to retain cytokines in the cytoplasm; (2) the cells have to be permeabilized prior to the staining of the cells with antibodies against the cytokines, which makes it impossible to expand the sorted T cells.

### MHC-multimer staining

MHC-multimers are synthetic structures made from HLA molecules linked together to form a multimeric complex which are loaded with antigen-specific peptide. Cells stained with multimer and antibodies against surface markers can be analyzed using a flow cytometry. The fluorescence intensity using the tetramer loaded with a high-avidity peptide derived from virus is usually high enough to discriminate the positive population in contrast to the result using the tetramer loaded with a low-avidity peptide derived from autologous antigen (54). A representative result is shown in Figure 1C.

The advantages of multimer assays are as follows: (1) combined analysis of phenotyping and specificity can be performed using the antibodies against surface markers. MHC-multimer can detect T cells which do not secrete cytokines, for instance naïve T cells. In combination with the phenotyping and MHC-multimer staining, we can assess the frequency of all antigen-specific T cells including dysfunctional/non-functional; (2) antigen-specific T cells can be sorted with a high purity. For this purpose, the streptamer technology is demonstrated to be useful (54, 55). Sorted cells can be used for adoptive T-cell therapy as a GMP-grade agent without regulatory issues (55).

The disadvantages of multimer staining are: (1) the multimer is not able to assess the functional status of antigen-specific T cells simultaneously. There can be a discrepancy in the frequency of antigen-specific T cells detected by multimer and by ELISPOT/ICS. Several papers reported that T cells detected by ICS were more important than those detected by multimer to control infectious diseases as demonstrated in the study of CMV infection (56–58). Multimer assays can be combined with functional assays, but it is well-known that the stimulation with a respective peptide leads to loss of multimer staining due to the downregulation of T-cell receptor, in particular when a high-avidity peptide is used (59); (2) MHC-multimer staining is HLA-specific and peptide-specific. Therefore the whole cell repertoire directed against a pathogen cannot yet be determined using MHC-multimer technology.

## Conclusion

T-cell monitoring against specific targets including viruses and fungi is ready to be integrated in the clinical practice. The monitoring of pathogen-specific T cells may help to define the individual MPE of a patient to develop a certain infections complication and to assess the potential benefit of adoptive T-cell therapy against certain pathogens.

## Conflict of Interest Statement

The authors declare that the research was conducted in the absence of any commercial or financial relationships that could be construed as a potential conflict of interest.
